# Evaluation of Strain Transition Properties between Cast-In Fibre Bragg Gratings and Cast Aluminium during Uniaxial Straining

**DOI:** 10.3390/s20216276

**Published:** 2020-11-04

**Authors:** Florian Heilmeier, Robert Koos, Michael Singer, Constantin Bauer, Peter Hornberger, Jochen Hiller, Wolfram Volk

**Affiliations:** 1 Chair of Metal Forming and Casting, Technical University of Munich (TUM), 85748 Garching, Germany; michael.singer@singeroelk.de (M.S.); cob@utg.de (C.B.); wv@utg.de (W.V.); 2 Research Neutron Source Heinz Maier-Leibnitz, TUM, 85748 Garching, Germany; robert.koos@frm2.tum.de; 3 Application Center for CT in Metrology, Fraunhofer Institute for Integrated Circuits, IIS, 94469 Deggendorf, Germany; peter.hornberger@th-deg.de; 4 Department of Mechanical Engineering and Mechatronics, Deggendorf Institute of Technology, 94469 Deggendorf, Germany; jochen.hiller@th-deg.de

**Keywords:** Fibre Bragg Gratings, neutron diffraction, X-ray tomography, tensile test

## Abstract

Current testing methods are capable of measuring strain near the surface on structural parts, for example by using strain gauges. However, stress peaks often occur within the material and can only be approximated. An alternative strain measurement incorporates fibre-optical strain sensors (Fiber Bragg Gratings, FBG) which are able to determine strains within the material. The principle has already been verified by using embedded FBGs in tensile specimens. The transition area between fibre and aluminium, however, is not yet properly investigated. Therefore, strains in tensile specimens containing FBGs were measured by neutron diffraction in gauge volumes of two different sizes around the Bragg grating. As a result, it is possible to identify and decouple elastic and plastic strains affecting the FBGs and to transfer the findings into a fully descriptive FE-model of the strain transition area.We thus accomplished closing the gap between the external load and internal straining obtained from cast-in FBG and generating valuable information about the mechanisms within the strain transition area.It was found that the porosity within the casting has a significant impact on the stiffness of the tensile specimen, the generation of excess microscopic tensions and thus the formation of permanent plastic strains, which are well recognized by the FBG. The knowledge that FBG as internal strain sensors function just as well as common external strain sensors will now allow for the application of FBG in actual structural parts and measurements under real load conditions. In the future, applications for long-term monitoring of cast parts will also be enabled and are currently under development.

## 1. Introduction

A precise understanding of material behaviour is essential for the load-specific design of structural components. Although there is much effort involved in designing structural parts, exact data for local strains and stresses under load are often unavailable. Consequently, the compounds are oversized by design, in order to ensure that they do not fail under normal load conditions [1]. The use of advanced internal measurement methods now provides a means to obtain valid strain information in structural parts under operating conditions and is therefore a promising approach to address this problem. Fibre-optical strain sensors (Fibre Bragg Gratings, FBGs) are strain sensors which can be cast into aluminium parts, as already shown by Weraneck et al. [2]. At the current state of development, structural parts are commonly monitored by the use of FBGs. In addition to using FBG as a substitute for strain gauges, the trend in research and development is to use component-integrated sensors based on FBG. There are several interesting applications of this method. Due to the low diameter of the glass fibre FBGs are being put into fibre reinforced plastics [3]. One application of embedded FBGs is the possibility to record strains during the curing of epoxy resin matrix. The associated knowledge of process-related residual stresses has particular advantages in manufacturing fibre-reinforced laminated metals [4]. FBG sensors integrated into fibre reinforced plastics are often used to examine impairments and delaminations by Low-Velocity-Impacts [5]. This principle is also applicable to steel cables with cores consisting of fibre reinforced plastics used for bridge constructions. This has the advantage of long-term strain measurements due to the inserted FBG [6]. Thus, FBG sensors can also be used in the construction industry. A further application proposes the use of FBG as a humidity sensor in composites of wooden bridges. Since wood swells under humidity, FBG strain measurements allow for conclusions about the humidity value and an assessment of the damages in the outer structure of the bridge [7]. FBGs are also applied to monitor the progression of corrosion of construction steels [8]. This is possible due to the resistance of glass fibres against corrosive. This quality is also beneficial for applications in the oil industry, where Zhou et al. describes FBGs as being used as pressure sensors within boreholes [9]. To summarize, FBGs have a lot of advantages and thus can be applied to a vast variety of measurement applications.

**Figure 1:**
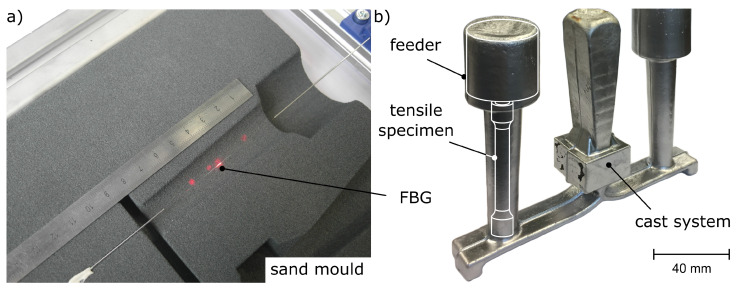
(**a**) One half of the instrumented mould with highlighted FBG. (**b**) Unfinished casting with cast system and two specimens each. After machining, the standard tensile specimen according to [12] contains a fully functional FBG. According to [13].

In this work, we utilized the small diameter and the resistance of glass fibres against the corrosive effect of aluminium melts to cast FBGs into aluminium parts, see Figure 1a). By doing this, they function as internal strain sensors (Figure 1b)). This measurement principle has been applied to cast tensile specimens made from the hypoeutectic cast alloy AlSi9Cu3 at utg. First calibration efforts, conducted by comparing strain measurements on the inside as well as on the outside of tensile specimens [10], showed that the calibration factor of the FBG differs from the factor obtained from the free fibres as determined by Jülich et al. [11].

In the effort to calibrate cast-in FBGs during casting [13] and uniaxial straining [10], Heilmeier et al. shows a wide dispersion of calibration factors. Obviously, cast-in FBGs behave differently compared to free FBGs, which can be precisely calibrated. In general, embedded FBGs can be loaded with axial and transversal strains according to [14]. Thus, for a better understanding of the strain transfer mechanisms from aluminium to fibre, the transition area between FBG and surrounding aluminium needs to be investigated in axial and transversal direction. To achieve this, the local microstrains within the contributing phases need to be obtained by neutron diffraction on two different volume scales to identify their influence on the strain measurement by FBGs. As a result, we will be able to identify and decouple elastic and plastic strains affecting the FBGs. This will allow the application of internal strain sensors in actual structural parts and subsequently, measurements under real load conditions.

## 2. Materials and Methods

### 2.1. Fibre Bragg Gratings

**Figure 2:**
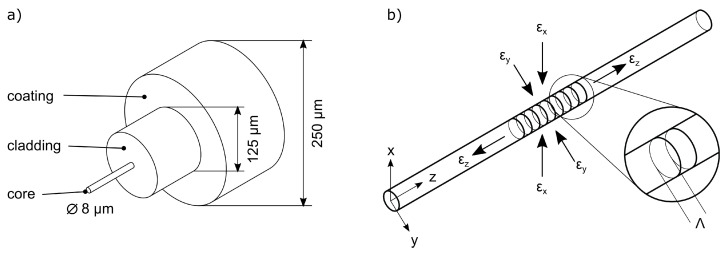
(**a**) Structure of a single-mode SMF28 glass fibre. The resulting diameter after decoating is 125μm. (**b**) Inner structure of a Bragg grating within the fibre’s core. The embedded fibre can be impinged by longitudinal ($\epsilon_{z}$) and transversal strains ($\epsilon_{x}$, $\epsilon_{y}$).

The following section describes the measurement method that was used. An FBG is a periodic change in the refraction index within the core of a glass fibre. Thus, a strain sensitive area is given along the fibre’s axis, which is measured by an optical interrogator. For the internal strain measurements during tensile testing we used a 3 mm long femtosecond grating within an SMF28 glass fibre, which can be cast into aluminium alloys [2]. According to the findings of Heilmeier et al., the fibre has a force-locked connection to the surrounding casting [10]. Figure 2a shows a sketch of a single mode fibre with a core diameter of 8μm. The coating is removed before any further processing leading to an effective fibre diameter of 125μm. The grating within the fibre’s core is depicted in Figure 2b. This figure shows that the grating is affected by both the axial strain $\epsilon_{z}$ and the transversal strains $\epsilon_{x}$ and $\epsilon_{y}$.

The absolute Bragg wavelength $\lambda_{B}$ of the reflected spectrum is given by

(1) $$\lambda_{B}=2n_{eff}\Lambda$$

and depends on the grating period Λ as well as the effective refractive index $n_{eff}$ [15]. Changes in Λ, by external straining, lead to a shift $\Delta \lambda_{B}$ of the peak wavelength according to [16]:

(2) $$\Delta \lambda_{B}=\lambda_{B,0}\left(1-p_{e}\right)\epsilon_{z}$$

Here, $\lambda_{B,0}$ is the initial peak wavelength of the free fibre without external straining and $p_{e}$ is the effective photoelastic constant. For embedded FBGs, the strain $\epsilon_{z}$ can cause transversal strains by the transversal contraction of the surrounding material. In this case, Equation (2) expands to

(3) $$\frac{\Delta \lambda_{B,x}}{\lambda_{B,0}}=\epsilon_{z}-\frac{n_{0}^{2}}{2}\left[p_{11}\epsilon_{x}+p_{12}\left(\epsilon_{y}+\epsilon_{z}\right)\right]$$

(4) $$\frac{\Delta \lambda_{B,y}}{\lambda_{B,0}}=\epsilon_{z}-\frac{n_{0}^{2}}{2}\left[p_{11}\epsilon_{y}+p_{12}\left(\epsilon_{x}+\epsilon_{z}\right)\right]$$

for the x- and y-direction [17]. Here, local photoelastic constants $p_{11}$ and $p_{12}$ depend on the direction which is currently referred to. Due to birefringence, there may be more than one distinct peak within the FBG’s spectrum. This is why we use a peakfinding algorithm which tracks the primary peak according to Heilmeier et al. The primary peak originates from the initial peak of the FBG and represents the axial strain $\epsilon_{z}$ during tensile testing [10].

We chose femtosecond FBGs because of their thermal stability. In our recent work, we found that the gratings withstand cast temperatures up to 750 °C without an excess degradation of their spectra. The resulting reflectivity of at least 50% of the initial intensity grants robust measurements during and after casting [14]. Table 1 shows the main FBG properties, including the conversion factor k=0.795 of the free fibre, which we used for the evaluation of $\epsilon_{z}$ according to Equation (5) [18]:

(5) $$\frac{\Delta \lambda }{\lambda_{B}}=k\cdot \epsilon_{z}$$

We found that this approach is valid, if only the primary peak is evaluated.

### 2.2. Cast Materials

The standardized hypoeutectic cast alloy AlSi9Cu3(Fe) [12] was used to cast the specimens for this investigation. It is commonly used for the production of structural parts using sand moulds, die casting and high pressure die casting. For grain refinement we used an aluminium-titanium boride (Al-TiB_2_) master alloy [19] to ensure better grain statistics during neutron diffraction [20]. The actual composition of the cast material is shown in Table 2. The characterization using the specimens after testing was conducted by spark emission spectroscopy.

**Figure 3:**
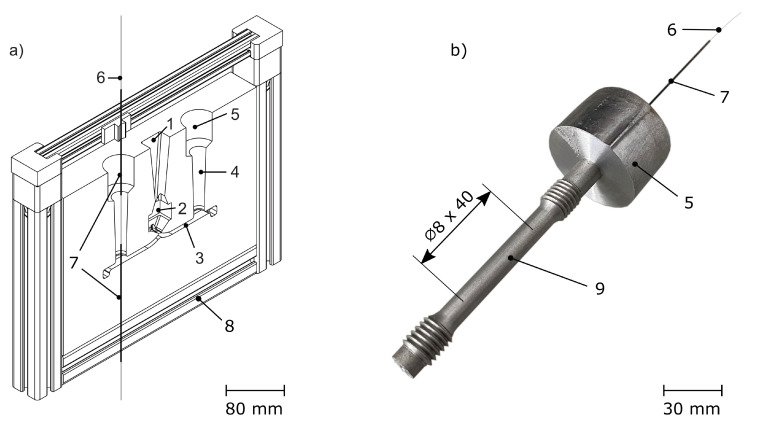
(**a**) One half of the cast mould. The melt is poured into the inlet (1), passes the filter (2) and is split by the runner (3). This way, two specimens (4) with feeders (5) on top can be cast simultaneously. The fibre (6) is protected by steel capillaries (7), which are supported by a frame (8). (**b**) Tensile specimen (9) after machining, containing a fully functional internal FBG.

For casting we used 3D-printed furan resin-bound silica sand moulds, from which one half is shown in Figure 3a. The melt is poured into the inlet at a cast temperature of 700 °C and is split by a runner after passing through the filter. This way, two specimens, each containing an FBG, can be cast. During machining, the feeder remains on the specimen. This way, the fibre is not harmed during machining and the specimen contains a fully functional FBG for tensile testing—see Figure 3b.

**Figure 4:**
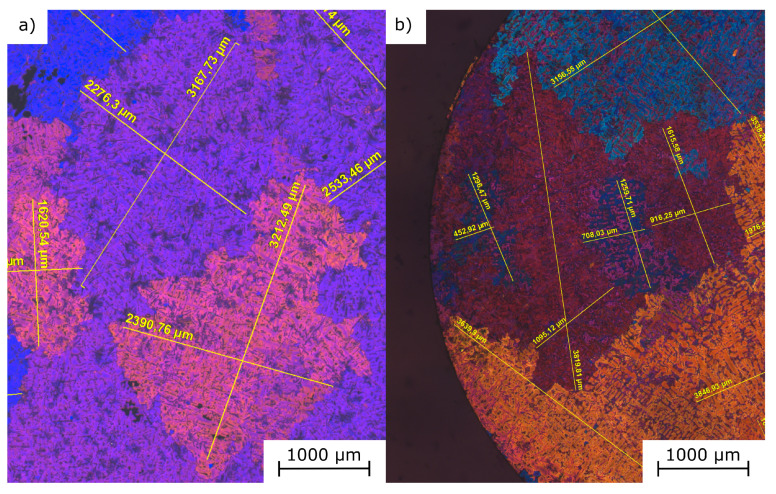
Images obtained from the dark field microscopy of etched aluminium micrographs. The effect of grain refinement of titanium boride in (**b**) leads to a finer microstructure compared to non-grain refined aluminium in (**a**) and thus to a higher stiffness of the tensile specimens.

The effect of grain refinement is shown in Figure 4. The dark field microscopy shows the texture of the etched micrographs with distinct areas of different colours. Each area represents one grain of the cast alloy, which is measured in directions of the large and small half axis. The mean values of (a) 3260μm versus 2040μm and (b) 2200μm versus 1180μm, respectively, show a significant reduction in the grain sizes, which leads to a stiffer material. This effect is described in Section 3.2.

### 2.3. Neutron Diffraction

**Figure 5:**
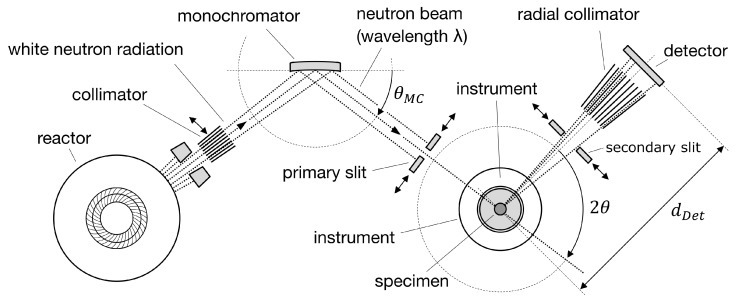
Fundamental setup of the neutron diffractometer STRESS-SPEC [23].

Neutron diffraction is a common method for destruction-free measurement of the internal straining of crystalline materials. At the research neutron source FRM2 (TUM) in Garching [21], the neutron flux is generated by nuclear fission of ^235^U in a water-moderated chain reaction which emits white neutron radiation [22]. After being reflected by a Si400 monochromator at a wavelength of $1.67\;\overset{˚}{A}$, the monochromatic neutron beam passes through the primary slit and penetrates the specimen, where it is diffracted by the hkl-lattice planes. After passing through a secondary slit, followed by a radial collimator, the diffracted beam is detected—see Figure 5. The gauge volume inside the specimen is defined by the height and width of the primary beam in combination with the width of the secondary slit [23].

Neutron diffraction as a measurement technique is based on the scattering of neutrons by the lattice planes of crystallines materials. The resulting path difference causes an interference, which was first defined by W. H. Bragg as a fundamental equation [24] as follows:

(6) $$2d_{hkl}\;sin\theta_{hkl}=\lambda$$

**Figure 6:**
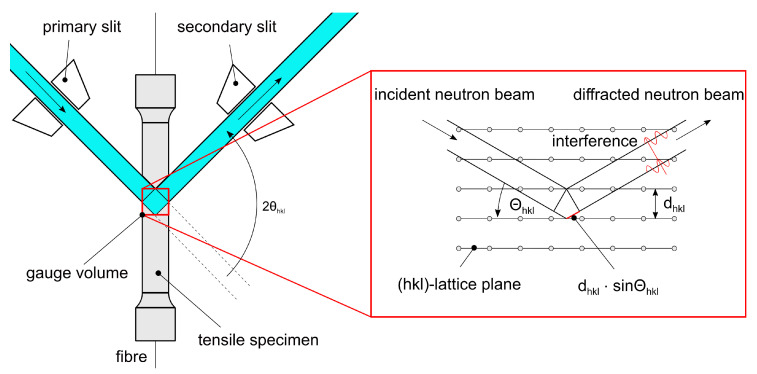
Principle of neutron diffraction, illustrated by the gauge volume within a tensile specimen. The incident neutron beam is diffracted by the hkl-lattice planes of the α-crystallites, according to Equation (6).

The measurement principle is illustrated in Figure 6. The gauge volume exactly matches the middle of the tensile specimen, where the internal FBG is situated. The diffraction angle $2\theta_{hkl}$ is extracted from the detector images. The change in $2\theta_{hkl}$ results from the change in $d_{hkl}$ during external straining and serves as basis for the calculation of the lattice spacing by using Equation (7) [25].

(7) $$\epsilon_{hkl}=\frac{d_{\sigma ,hkl}-d_{0,hkl}}{d_{0,hkl}}=\frac{sin\Theta_{0,hkl}}{sin\Theta_{\sigma ,hkl}}-1.$$

### 2.4. X-ray Computed Tomography

X-ray computed tomography (CT) is an imaging technique, which is increasingly used as a powerful, non destructive tool for visualizing 3D micro-structures. It enjoys enormous popularity in research and development, especially for material science applications due to its ability to achieve volumetric image resolutions at micrometer scale [26].

**Figure 7:**
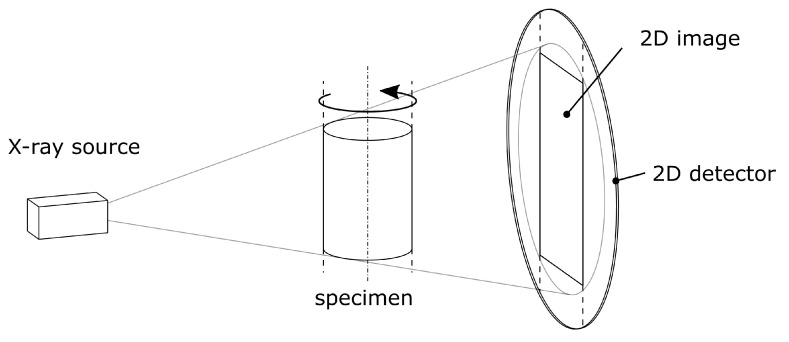
Schematic representation of the cone-beam CT imaging technique which gives evidence of the inner structure of the specimen, such as porosity. According to [29].

The cone-beam CT represents a state of the art scanning principle [27] which is depicted in Figure 7. Multiple 2D X-ray projection images are taken from different angles, enabled by a rotation of the scanned object within the X-ray cone beam. The flat photon detector is used to digitize the projections in form of grey value coded images for further processing.

The principle is based on the partial attenuation of the X-ray beam by matter, following an exponential law. Longer penetration depths lead to darker areas on the projected image. The absorption also increases with both higher density and atomic number, while it decreases with higher photon energies.

With the total amount of images taken, the inner structure of the sample can be determined by mathematical 3D reconstruction methods [28]. For this reason, we used the cone-beam algorithm by Feldkamp, Davis and Kress [29].

### 2.5. Mathematical Operations

All measured strains depicted in Section 4 are fitted by using the empirical, mathematical description of flow curves by Ludwik-Hollomon, according to [30]:

(8) $$\sigma_{\phi }=k\cdot \phi^{n}+\sigma_{0}$$

where $\sigma_{\phi }$ is the yield stress and ϕ the degree of deformation. $\sigma_{0}$ considers a possible prestress on the specimen. This basic exponentional term is used to smooth the measured data points for a direct comparability of the experimental variants. The root mean squared error obtained by fitting Equation (8) to the measured data is propagated as an additional error value in combination with the errors of the measured data. An estimation of the resulting errors is given by the square root of the summed squared individual errors $s_{\epsilon ,i}$, according to Gauss [31]

(9) $$s_{\epsilon ,res}=\sqrt{\sum_{i=1}^{n}s_{\epsilon ,i}^{2}}$$

## 3. Experimental Setup and Simulation Model

In order to evaluate the strain transition between cast-in FBGs and surrounding aluminium, we simultaneously measured the internal straining obtained from FBG and neutron diffraction as well as the external strain with an extensometer during unixaial tensile testing. The measurements are accompanied by a simulation, which models the cast-in FBG under external loads. The employed methods are described below.

### 3.1. Experimental Setup

**Figure 8:**
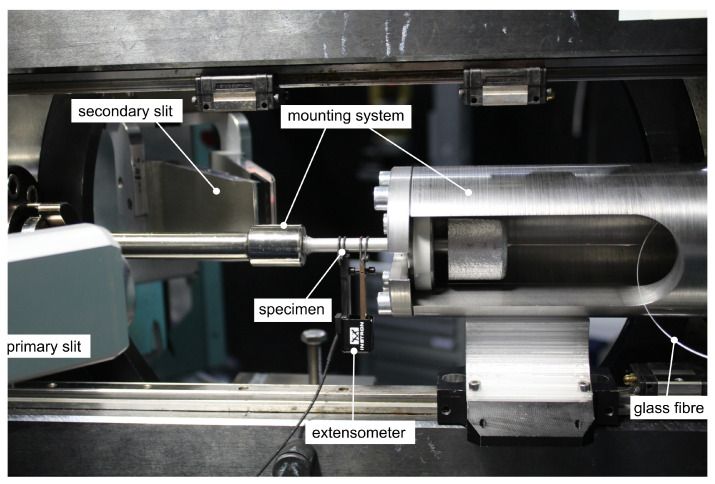
Experimental setup on site for the STRESS-SPEC instrument for simultaneous strain measurements using FBG and neutron diffraction. The primary and secondary slits are adjusted automatically to enable two different gauge volumes during testing. There is an additional extensometer which is situated directly on the specimen.

The experimental setup for internal strain measurements using FBGs has been established onsite the STRESS-SPEC instrument [32] at the research neutron source FRM2 (TUM) in Garching [21] as shown in Figure 8. The setup incorporates a mounting system, which allows the feeder to remain on the tensile specimen. This is of particular importance because the FBG requires a functional connection to the measurement equipment.

The strain of the specimens was measured in situ and ex situ by extensometer, FBGs and neutron diffraction simultaneously using the tensile rig at STRESS-SPEC [33]. The neutron beam covered two differently sized gauge volumes (0.5×8×8 mm^3^ and 0.5×0.5×8 mm^3^) formed by a 0.5 mm collimator and an automatically adjustable primary slit. A wavelength of $\lambda =1.67\;\overset{˚}{A}$ was used for acquiring the diffraction peaks of Al(311) at 2*θ* = 86° and a scan time of 300 s. The specimens rotated around the vertical axis to improve grain statistics. The primary and secondary slit are directed to the middle of the specimen and form a gauge volume which is congruent to the FBG and its measurement direction along the axis of the specimen. Figure 6 shows the measurement principle on a microscopic scale. The incident neutron beam is diffracted by the lattices planes of the α-crystallites within the casting, which meet the Bragg relation in Equation (6) at an angle of 2*θ* ∼ 86°.

During the experiment, the specimens were loaded with increasing load steps (in situ), subsequently followed by a release of the force (ex situ). The load steps are shown in Table 3. For a sufficient neutron count rate by the detector, each step with big volume lasts 480 s, whereas the small volumes have a scan time of 900 s.

### 3.2. Simulation

**Figure 9:**
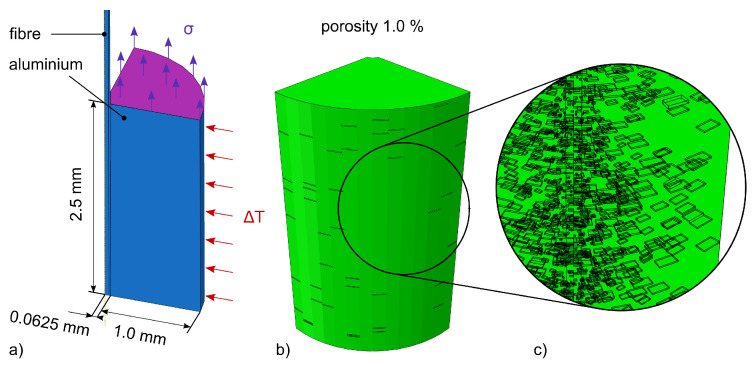
(**a**) Sketch of the simulation model consisting of fibre with surrounding aluminium. Due to symmetry, the model represents one eighth of the whole body. The two boundary conditions ΔT and σ represent two consecutive steps of the straining depicted in Figure 10. (**b**) Mesh of the model with a porosity of 1.0 vol.-%. (**c**) Detailed view on the void distribution within the model.

**Figure 10:**
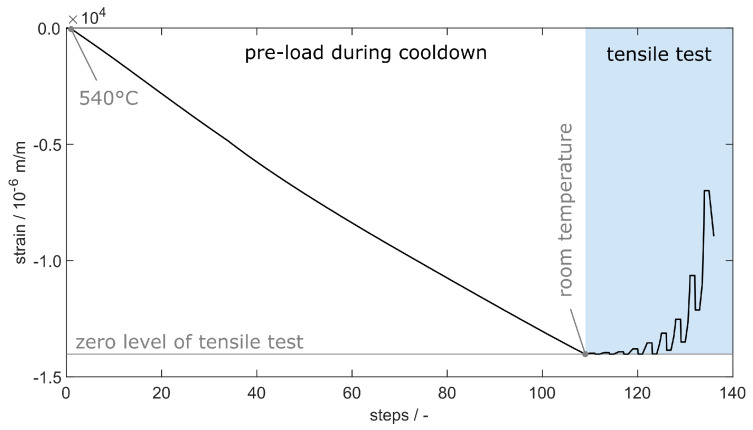
Definition of the simulation steps with explicit differentiation between the pre-load and tensile test. The pre-load according to [13] leads to a realistic force-locked connection between aluminium and fibre as a start condition for the subsequent tensile test at room temperature.

For a distinct evaluation of the strain transition from aluminium to fibre, we used the implicit finite element (FE) simulation given in Figure 9, as already presented in [13]. In order to model the interaction properly, the simulation starts with the cooling of the aluminium body, which forces compression strains onto the fibre—see Figure 10. The resulting force-locked connection is used as a start condition for the subsequent simulation of the tensile test with increasing load steps, as listed in Table 3. We extended the model by defining a step-wise increasing load σ onto the upper surface of the aluminium body as depicted in Figure 9a.

For an additional evaluation on how the occurring porosity within the specimen affects the strain transition, the aluminium body contains voids, which are simulated by a random deletion of nodes in the FE-mesh, as already described by Heilmeier et al. [13]. The mesh with deleted nodes is shown in Figure 9b, while Figure 9c shows a detailed view of the voids on the model’s surface.

The material model we used is based on a series of hot tensile tests, which were conducted by Reihle [34]. The resulting temperature-resolved Mises-yield surface represents the macroscopic material behaviour. The cast material tested by Reihle was not grain refined like the one in this survey, which is why we calibrated the simulation using the in situ strain response measured by FBG. The yield strength at room temperature has been extended to 104% of its original stiffness to match load step 9 in Figure 14b.

### 3.3. Porosity Evaluation by Computed Tomography

**Figure 11:**
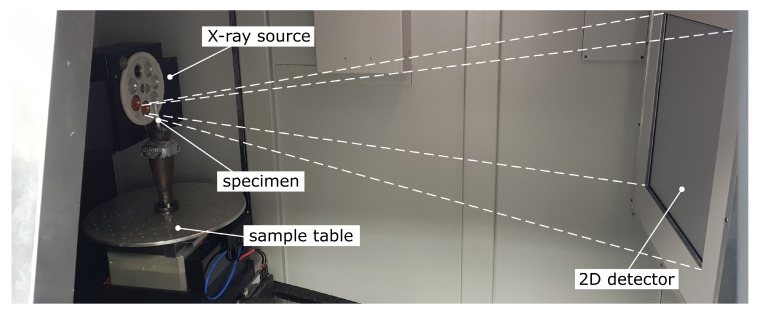
The micro-focus CT-scanner Tomoscope HV 500 from Werth Messtechnik GmbH with the tensile specimen, positioned directly in front of the X-ray source for high image magnification.

In order to perform a porosity analysis, high-resolution 3D-image data were obtained from the tensile specimen using industrial computed tomography. The corresponding scanner is equipped with a micro-focus X-ray source, which illuminates a 2k-detector with a pixel pitch of 200μm. The scanning region in the middle of the sample comprises the complete diameter by a scan height of 10 mm. Due to this small region, a high magnification (compare with Figure 11) and thus a high volume resolution of 8μm is achieved at a scanning time of 90 min and a maximum X-ray energy of 170 keV. In order to avoid artefacts arising from the polychromatic characteristic of the spectrum, the spectrum is pre-filtered by a copper plate of 1 mm thickness [35].

Since materials can be distinguished in the volume, a porosity evaluation is possible, as long as they have different absorption coefficients. For evaluation, the VGDefX algorithm was used, which is part of the porosity/inclusion analysis module of VG Studio Max [36]. This algorithm offers a reliable pore detection and indicates the probability of occurrence for a statistic evaluation. It was used because it takes grey value variations into account and applies noise reduction. In the analysis, an automatic surface determination with local thresholding is used.

## 4. Results

In this investigation, we conducted tensile tests with three independent strain measurement techniques. One of them is the new approach of optical strain measurements with cast-in FBGs. The CT-scan gives evidence of the porosity within the specimens with an overall evaluation using 3D-image-reconstruction methods. We will present specimens FS54 and FS55 as well as ZS25 and ZS26, which show less and more porosity, respectively. Both variants are fitted by Equation (8) for a generalization of the strain progressions. All tests are accompanied by an FE-simulation, which is used for the final evaluation of the strain transition from aluminium to fibre.

### 4.1. Porosity Evaluation

**Figure 12:**
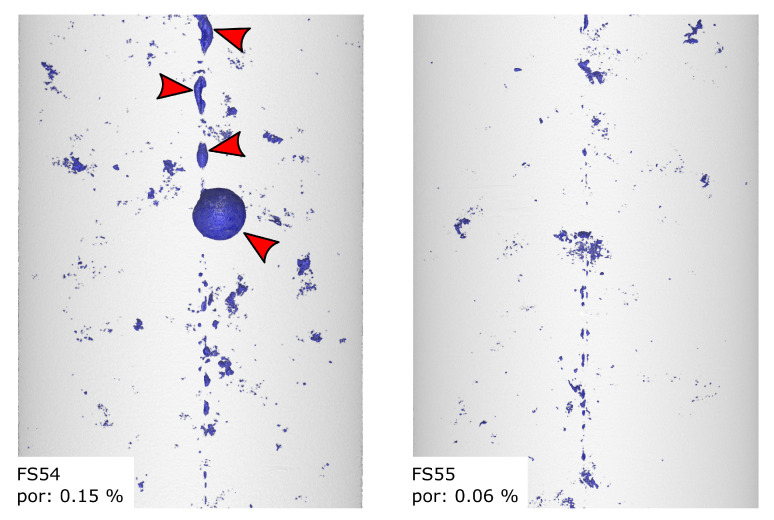
CT scans of specimens FS54 and FS55. The microstructure shows only a small amount of micro blowholes and only occasional entrained gas bubbles (marked by arrows). This leads to an overall porosity values of 0.15 vol.-% and 0.06 vol.-%.

**Figure 13:**
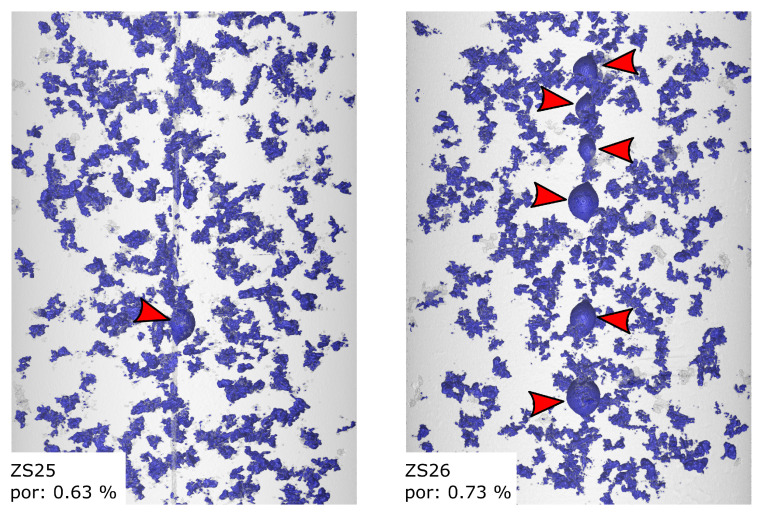
CT scans of specimens ZS25 and ZS26. These specimens were cast on another day leading to a much higher amount of micro blowholes as well as more entrained gas bubbles on the fibres marked by arrows. The resulting overall porosity values of 0.63 vol.-% and 0.73 vol.-% lead to a weaker microstructure.

Figure 12 shows the micro CT-scan of specimens FS54 and FS55 with porosity values of 0.15% and 0.06%, which are rather small for AlSi9Cu3(Fe) cast into sand moulds. The majority of the occurring voids is formed by microscopic blowholes with occasional bubbles of entrained gas held onto the glass fibres, as can be seen marked by the red arrows. In contrast, ZS25 and ZS26 show higher porosity values of 0.63% and 0.73%, which are formed by the very prominent micro blowholes and the more frequent occurrence of entrained gas bubbles on the glass fibres. The air bubbles are marked by red arrows in Figure 13. These specimens were cast on a different day, leading to different cast conditions, such as ambient temperature and humidity.

### 4.2. Strain Evaluation during Tensile Testing

The tensile tests enable the comparison of three independent strain measurement techniques. The extensometer data represent the macroscopic behaviour of the specimens under external load. The cast-in FBGs provide strain information from within the specimens. The phase-specific straining is given by neutron diffraction with two different gauge volumes around the fibre. Each strain evolution consists of two data sets measured by specimens obtained from one casting, which were unified using a fitting curve given by Equation (8).

**Figure 14:**
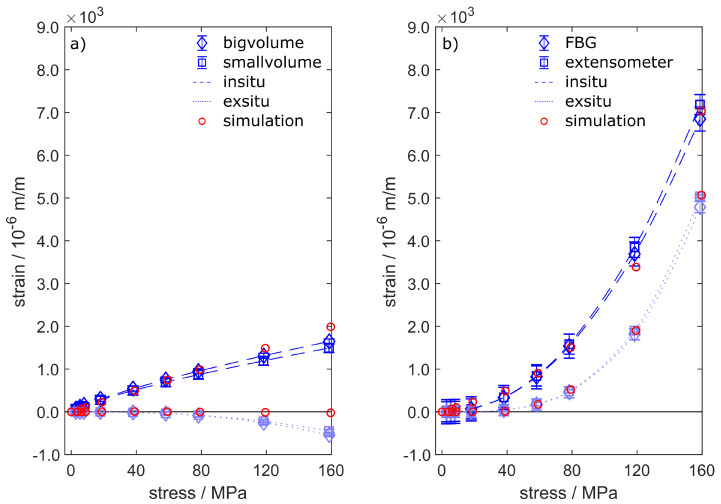
Straining of specimens FS54 and FS55 under tensile load steps until fatigue. The figure shows both the comparison of in situ and ex situ data obtained by (**a**) neutron diffraction and (**b**) by extensometer and FBG. The simulation does not consider the microstructure of the aluminium and thus the microscopic straining does not show any permanent strains. Besides that, the calculated data in (**b**) match the measured strains well. The measured strain reactions have been fitted by Equation (8).

The tensile test routine presented in Table 3 leads to strain reactions, which are shown in Figure 14 for FS54 and FS55. Figure 14a depicts the phase-specific strains within the Al311-crystallites. AlSi alloys commonly show heterogeneous microstructures, which combines stiff Si particles with ductile α-aluminium into a composite with a combined strength, see Schöbel et al. [37]. Due to the ductility of the α-aluminium, which is measured specifically by neutron diffraction in form of the Al311-reflection, the measured strains in Figure 14a are smaller than the macroscopic strains depicted in Figure 14b. The big and small gauge volumes both show similar in situ strain reactions, whereby the small volume generates slightly smaller values. The similar strains can be explained by the small amount of porosity as depicted by Figure 12, leading to an even strain distribution over the gauge volumes. The simulated strain data show very good agreement with the measured data, where only the last two load steps are overvalued by up to $340\times 10^{-6}$ m/m. The ex situ data of Al311 show compressive strains, which is a microscopic reaction to the presence of silicon-rich precipitations within the alloy. Due to the homogeneous material model within the simulation, this effect cannot be accurately recreated and thus the elastic strains take on a value of zero when the force is relieved.

The straining obtained from FBG and extensometer is depicted in Figure 14b. The data show good agreement between FBG and extensometer, meaning that cast-in FBG are perfectly capable of measuring precise strain data conforming to standards [38]. The maximum in situ straining shows values as high as $7000\times 10^{-6}$ m/m. This data point is used to calibrate the simulation model, from which all other calculated strain values arise.

The ex-situ straining shows the plastification of the specimens, beginning at tensions higher than 40 MPa. Both extensometer and FBG recognize plastic strains in the form of a permanent deformation, which remains after strain relief as ex situ straining. Here, again, the calculated strain data perfectly match the measured data. Due to the small amount of porosity within specimens FS54 and FS55, the strain calculations were obtained from the simulation model without porosity.

**Figure 15:**
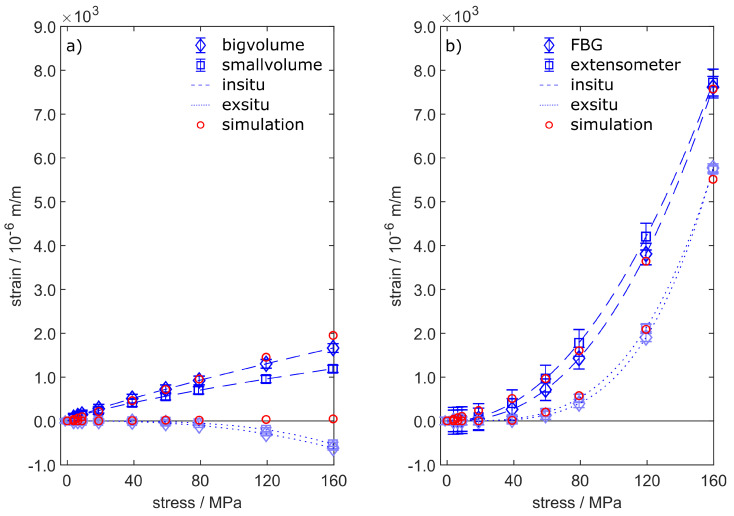
Straining of specimens ZS25 and ZS26 under tensile load steps until fatigue. The figure shows both the comparison of in situ and ex situ data obtained by (**a**) neutron diffraction and (**b**) by extensometer and FBG. The calculated data include 1.0 vol.-% porosity and match the measured strains well. The measured strain reactions have been fitted by Equation (8).

Concerning specimens ZS25 and ZS26, the best fitting results were calculated by adding 1.0% porosity to the model as described in Section 3.2. The resulting quality was evaluated with respect to the highest in situ strain value in Figure 15b. Again, the model does not take the microstructure of the alloy into account. Thus, the phase-specific straining of Al311 is overrated for the in situ steps and takes on zero-values for the ex situ steps. The in situ and ex situ straining of the fibre and the extensometer, which again show very good agreement to each other, are perfectly matched by the simulation.

In conclusion, the results of the simulation model presented in Section 3.2 could be verified by all the different strain measurement techniques we used during tensile testing. Especially the phase-specific strain data obtained by neutron diffraction provides valuable support, leading to a fully descriptive model of the interaction between glass fibre and cast aluminium. Based on these findings, we are now able to examine the strain transition from aluminium to fibre as a function of the radius on a microscopic scale.

### 4.3. Evaluation of the Strain Transition Area

**Figure 16:**
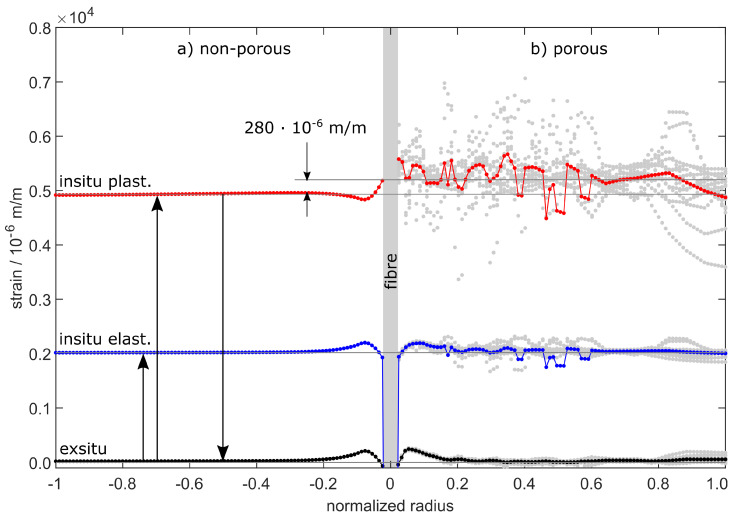
Strain transition area as obtained from the calibrated simulation model. The straining versus normalized radius shows the in situ elastic and plastic strains as well as the ex situ strains for (**a**) the non-porous model and (**b**) the model with 1.0 vol.-% porosity.

The simulation model now allows for a closer look at the strain transition area as a function of the model’s radius. Therefore, Figure 16 shows the strain distribution for load step 9 at 160 MPa, according to Table 3. The radius is given by normalized values beginning from zero in the middle, where the fibre is indicated, to the fringe of the aluminium.

In Figure 16 the non-porous material to the left is compared to the material with 1.0 vol.-% porosity to the right. The in situ elastic straining shows $2000\times 10^{-6}$ m/m for both models, which is in compliance to the results in Figure 14a and Figure 15a. In addition, the plastic straining is given, which is generated during the in situ steps and remains as permanent straining when the force is relieved.

After relief, the elastic ex situ strains return to zero except for the immediate surrounding of the fibre. Note that the data points deposited in grey colour are the direct calculations of the porous model. As the model is speckled with voids, the results are not radially symmetrical any more, which is why we calculated the mean values versus radius.

Naturally, the voids within the aluminium are not able to transfer stresses, which weakens the whole material by the generation of local excessive tensions. This leads to increased overall plastic strains beginning at tensions higher than 40 MPa. The plastic strains sum up over the entirety of all in situ load steps leading to higher measured values in Figure 15b than in Figure 14b. This does not affect the elastic share of the overall straining, which solely depends on the yield stress given by the material model. This is why the elastic strain progressions for both models share a common mean value in Figure 16.

**Figure 17:**
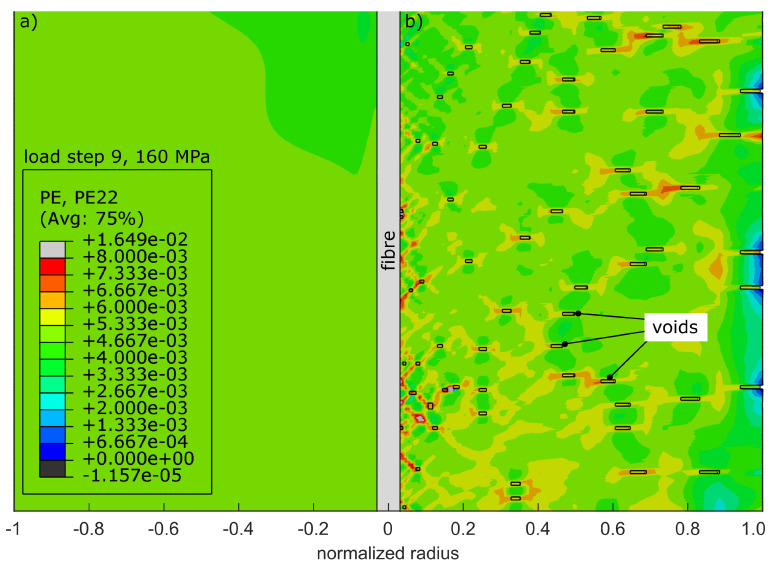
Microscopic plastic straining in the transition area around the glass fibre (**a**) without and (**b**) with 1.0 vol.-% porosity. By adding voids to the model, local excessive tensions are formed, weakening the structure of the specimen.

Due to the voids, local excessive tensions are forming within the aluminium. The related plastic strains are depicted in Figure 17b in direct comparison to the plastic strains in the absence of the voids (Figure 17a). The voids are statistically allocated, which is why the effect of excessive tensions averages itself. The glass fibre is only affected by changing transversal tensions, which can cause a change in the FBG’s spectrum [17]. According to Heilmeier et al. [10], this has no influence on the FBG’s axial straining as long as only the primary peak is evaluated.

### 4.4. Spectra Analysis

**Figure 18:**
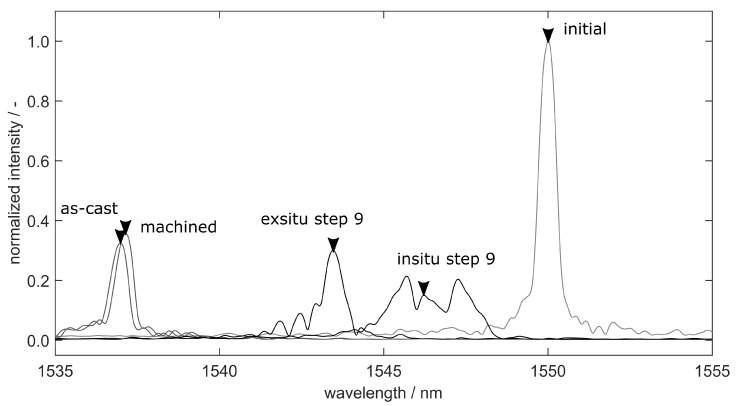
FBG-spectra obtained from every intermediate condition of the specimen before and during testing. The spectrum shows a decrease in reflectivity and degenerates with an increased load subsequently, where secondary peaks are formed beside the primary peak (marked by a black arrow for each step).

Figure 18 elucidates the alteration of the FBG’s spectrum. The position of the primary peak determined by the peakfinding algorithm is given by the black arrows. We chose specimen FS55 due to the small defect content (see Figure 12) in order to keep the influence of porosities on the spectrum at a minimum. All spectra are normalized using the initial peak, which is the given by the free fibre before casting. The peak intensity after casting is beneath 40% of its original intensity. The spectrum of the FBG after machining shows a relief of the force on the fibre, leading to a moderate rise in the peak intensity and a shift back to higher wavelengths. This is the initial state of the FBG before tensile testing.

In order to show the effect of the external force during tensile testing, the figure shows the spectrum which forms during the last load step and after relief. During in situ step 9, the spectrum shows distinct secondary peaks, which may be caused by transversal loads, strain gradients along the Bragg grating [17] or changes in the fibre’s local photoelastic constants—see Equations (3) and (4).

The peakfinding algorithm is designed to track the primary peak, which represents the axial straining $\epsilon_{z}$ of the grating. This was already proven by Heilmeier et al. [10] and can be directly seen by the agreement of the strain measurements in Figure 14b and Figure 15b. The cause of the occurring secondary peaks, however, has to be determined by further experiments. After relief, the spectrum obviously reshapes to a less distorted peak. This may be a proof for the secondary peaks to be caused by transversal strains, as the lateral contraction of the tensile specimen reduces after relief.

## 5. Discussion

The calculations show that increased plastic strains are generated by the voids within the aluminium due to local excessive tensions. This greatly affects the macroscopic behaviour of the specimens and thus the strain measurements by extensiometer and FBG—see Figure 14b and Figure 15b. Apparently, the microscopic strain distribution only affects the aluminium, as the glass fibre only shows changes in macroscopic strains. Obviously, the casting’s supporting effect is still granted in the presence of pores, as the glass fibre would otherwise have breached.

Above a tension of 40 MPa, plastic strains are generated, which build up during the in situ steps. The total plastic strain is then retained as total strain information within the glass fibre. Obviously, the strain transition between aluminium and fibre is not affected by the porosity. There is rather the effect of increased plastification of the specimens which, of course, cannot be directly detected during continuous tensile testing. Regarding [10], only in situ data were generated during testing, making it impossible to decouple plastic and elastic strains afterwards.

The wide range of porosity values between 0 vol.-% and 2 vol.-% can now explain the variation of calibration factors. This claim is sustained by [13], which states that the overall defect volume has a greater influence on the strain response of the cast-in FBG than the defect area directly on the fibre.

Nevertheless, the difference between strains measured by FBG and extensometer has not yet been clarified. This may be due to stick-slip effects, which we could not verify in our simulation.

Although the statement of the simulation is valid and provides quantitative results, the microscopic strain distribution highly depends on the shape and distribution of the voids within the model. This may have a small impact on the local microscopic strains, but sums up to a macroscopic difference in the overall straining and stiffness of the specimen. The validation in Figure 14 and Figure 15 shows excellent agreement between measurement and simulation and yields valuable information about the strain transition area. Thus, the FE-model closes the gap between microscopic and macroscopic straining. Of particular significance is the statement that cast-in FBG behaves like the external strain measurement method given by extensometer. The microstructure of the surrounding aluminium and the external load condition do affect the reflected spectra obtained from the Bragg grating. However, the strain measurement by FBG is not compromised if the primary peak is reliably tracked during the spectra analysis. Other than Lammens et al., who investigated transversal strains during the curation of cross-ply composites [39], we only focus on the distinct detection of the primary peak in this work, which represents the axial straining $\epsilon_{z}$. Nevertheless, if the occurring secondary peaks can be reliably related to transversal strains, the peak-finding method we used could be of certain interest for the evaluation of embedded FBGs.

## 6. Conclusions

In recent research, cast-in FBG showed a wide dispersion of calibration factors. In this research, we examined the question on how the strain transition from aluminium to fibre forms during tensile testing. Microscopic porosity has a great impact on the local microstrains generated. Both effects were evaluated by a calibrated finite element simulation, which is based on both the measurement of phase-specific strains using neutron diffraction and macrostrains by the extensometer, as well as FBG. As a result, we were able to identify and decouple elastic and plastic strains affecting the FBGs.

The experiments show that plastic strains develop at tensions higher than 40 MPa. Plastic strains remain permanently within the specimen and add up over all load steps. Because the fibre has a force-locked connection to the surrounding casting due to shrinkage after solidification, the FBG shows the same permanent straining in the plastic deformation regime of the specimen as the external strain measurement by extensometer.

Because of local excessive tensions in presence of pores regions more plastic straining is transferred into the FBG. This is shown by the FE simulation, which gives evidence of the local stress and strain distribution in the transition area, especially in the direct surroundings of the glass fibre. The content of porosity within the casting turns out to be the main influence on the straining of the fibre, which is then measured by the Bragg grating.

The porosity affects neither the calculated nor the measured phase-specific elastic strains of the aluminium crystallites. The mechanisms of plastification do not occur within the aluminium grains, but in between. This is why neutron diffraction is only able to measure elastic strains, which turn out to be independent from the pore content. This is substantiated by simulation, which is not able to show the microscopic, inter-granular ex situ strains within the aluminium, though. An interesting extension of the simulation model would hence consider a heterogeneous microstructure of the aluminium.

In conclusion, we accomplished to close the gap between the external load and internal straining obtained from cast-in FBG by development of a fully descriptive FE-model considering the contact between casting and glass fibre. This enables the generation of valuable information about the mechanisms within the strain transition obtained from the strain evolutions directly around the fibre. We see that the porosity has a significant impact on the stiffness of the tensile specimen, the generation of excess tensions and thus the formation of permanent plastic strains, which are well recognized by the FBG. The knowledge that FBG as internal strain sensors function just as common external strain sensors will allow the application of FBG in actual structural parts and measurements under real load conditions. In future, applications for long-term monitoring of cast parts will also be enabled and are currently under development.

## Figures and Tables

**Table 1:** Fibre properties.

Fibre Type	Single Mode SMF28
fibre diameter	125μm
grating type	femtosecond FBG
grating length	3 mm
initial wavelength $\lambda_{B,0}$	1550 nm
k-factor of the free fibre	0.795 [11]

**Table 2:** Standardized composition of AlSi9Cu3(Fe) and measured composition of grain refined AlSi9Cu3(Fe) obtained by spark emission spectroscopy.

(wt.%) Type	Si	Cu	Fe	Mn	Mg	Ti
AlSi9Cu3(Fe) standardized [12]	8.0–11.0	2.0–4.0	1.3	0.55	0.05–0.55	<0.20
AlSi9Cu3(Fe) as-cast and grain refined	9.1	3.1	0.74	0.28	0.21	0.010

**Table 3:** Increasing load steps during the tensile test. These represent the in situ load steps whereas the ex situ load steps are given by each subsequent force relief with identical measuring durations.

	Step 1	Step 2	Step 3	Step 4	Step 5	Step 6	Step 7	Step 8	Step 9
tension/MPa	5	7.5	10	20	40	60	80	120	160
time (big volume)/s	480	480	480	480	480	480	480	480	480
time (small volume)/s	900	900	900	900	900	900	900	900	900
